# Systems Biology of Microbial Infection

**DOI:** 10.3389/fmicb.2012.00328

**Published:** 2012-09-12

**Authors:** Reinhard Guthke, Jörg Linde, Franziska Mech, Marc Thilo Figge

**Affiliations:** ^1^Leibniz Institute for Natural Product Research and Infection Biology – Hans Knöll InstituteJena, Germany

This special Systems Biology of Microbial Infection research topic is dedicated to the dynamic modeling and model-driven analysis of microbial infection processes. It aims at describing and analyzing the confrontation of the host with bacterial and fungal pathogens, and at modeling and understanding the interactions of the immune system of humans and animals, with pathogens.

The early stages of the mathematical modeling of infectious diseases were initiated at the beginning of the twentieth century and were based on the population dynamics of epidemics, e.g., to design governmental vaccination policy (Ross, 1911, 1916).

Today, systems biology of microbial infection considers molecular, cellular, and even organismic levels of the pathogen and the host’s immune system and is directed toward personalized medicine and theranostics.

The presented papers comprise theoretical and experimental studies. Some contributions present an integrative analysis of genome-wide data from both the host and the pathogen. Other contributions report on spatio-temporal determinations of host-pathogen interactions or the response of the host and pathogenic cells to defined perturbations that simulate infectious conditions.

This research topic is organized in two parts, with the first part consisting of four review articles and the second part consisting of seven original research papers.

## Part I: Review Articles

Horn et al. (2012) review the systems biology of fungal infection, regarding virulence determinants of fungal pathogens, their adhesion and colonization of the host, their metabolism and gene expression patterns during infection, and their interaction with host cell types.

Haas (2012) reviews the regulation of iron uptake from the host and its storage within *Aspergillus fumigatus* cells as well as the regulation of iron homeostasis and transcriptional regulation under conditions of iron starvation within the pathogen.

Another example of adaptation to a pathogenic lifestyle to the conditions in the host is reviewed by Dandekar et al. (2012). The authors report on recent findings regarding the metabolic modeling of adaptation of *Salmonella* sp. to nutritional conditions within the host’s vacuoles and membrane-bound compartments. Furthermore, the required metabolic conditions during *Salmonella* infection are compared with those for *Listeria* and *Legionella*.

Next, infection arises if the pathogen multiplies and overgrows the normal microbial flora, either at the place of entrance or in deeper tissue layers and organs. This is reviewed by Fuchs et al. (2012) focusing on the bacterial replication of metabolism in *Listeria*.

## Part II: Original Research Papers

The original research papers demonstrate different systems biology approaches to model: (i) host-pathogen interactions, (ii) molecular interaction networks of the pathogen, and (iii) host responses to the microbial infection.

### Host-pathogen interaction

In the past, most studies have focused on either the side of the pathogen or the host. Tierney et al. (2012) try to overcome this limitation using Next Generation Sequencing (RNA-Seq analysis) to monitor the gene expression of the host and the pathogen simultaneously. They analyze and model the interaction of mouse bone marrow-derived dendritic cells with the pathogenic yeast *Candida albicans*, and infer a gene regulatory network comprising genes and proteins of both host and pathogen.

Durmuş Tekir et al. (2012) study more than 23,000 protein–protein interactions that characterize the strategies of interaction of the human host with viral, bacterial, and some fungal pathogens utilizing the database pathogen-host-search-interaction-tool (PHISTO).

Binder et al. (2012) modeled the population dynamics of *Borrelia* *burgdorferi* using an ordinary differential equation approach taking into account the dissemination of bacteria to different tissues as well as cellular phagocytic activity during the innate immune response. By comparing different model scenarios with experimental data on infected mice, they aim at understanding why an almost cleared population of these bacteria can recover and reach a size that is even larger than the initial population before entering the chronic phase.

Applying an agent-based modeling approach that treats cells as discrete objects in space with dynamic properties, Tokarski et al. (2012) simulated the interactions of conidia of the human-pathogenic fungus *A. fumigatus* with migrating human neutrophils. Based on the analysis of time-lapse microscopy image data, different scenarios of phagocyte migration are explored and evaluated with regard to the clearance efficiency.

### Molecular interaction network of the pathogen

A genome-wide gene regulatory network has been inferred by Altwasser et al. (2012) for *C. albicans* using a compendium of microarray data by integration of prior knowledge from different sources (e.g., transcription factor – target gene and protein–protein interaction data bases) as well as a large number of research papers screened for regulator/target gene interactions relations by an automatic text mining search.

### Host response to microbial infection

Monitoring the immune response by flow cytometry for immune cells and cytokine production, Simon et al. (2012) identified and analyzed correlations and decision rules that formulated multidimensional and non-linear relations between the measured data on T-helper-cells and the cytokines GM-CSF, IFN-gamma, IL-2, IL-17, RANKL, and TNF-alpha. The authors found that, in particular, the production of GM-CSF and IL-17 turned out to be highly correlated.

Translational systems biology aims at the integration of omics data and clinical data from individual patients based on the investigation of animal models for therapeutical interventions. Lambeck et al. (2012) analyzed and compared the transcriptome from blood samples of a murine sepsis model together with that of patients from the pediatric intensive care unit.

## Summary

Systems biology of microbial infection aims at the development of testable mathematical and computational models of host-pathogen interactions that have predictive power for diagnosis and therapy by focusing on biomarkers and drug targets. This Research Topic presents a survey on the current state of this field. Some results were already presented at the first International Workshop on the “Systems Biology of Microbial Infection” that was held in 2011 in Jena, Germany, and will be organized in a biennial fashion by the Leibniz Institute for Natural Product Research and Infection Biology – Hans Knöll Institute (HKI), Jena. Today, the field of systems biology of microbial infection is still in its infancy. However, we are convinced that the integration of “omics” approaches with image-based systems biology will strengthen and open new avenues to quantitative modeling of host-pathogen interactions in the future.
